# Positivity Rates and Serum Levels of Antibodies Against Histone H3 Lysine 9 and Its Lactylated Form in Rheumatoid Arthritis and Its Mimics

**DOI:** 10.3390/diagnostics16203305

**Published:** 2026-10-12

**Authors:** Hui-Chun Yu, Bao-Bao Hsu, Pin-Chen Chen, Ming-Chi Lu

**Affiliations:** 1 Department of Medical Research, Dalin Tzu Chi Hospital, Buddhist Tzu Chi Medical Foundation, Dalin, Chiayi 622401, Taiwan; 2 Division of Allergy, Immunology and Rheumatology, Dalin Tzu Chi Hospital, Buddhist Tzu Chi Medical Foundation, Dalin, Chiayi 622401, Taiwan; 3 School of Medicine, Tzu Chi University, Hualien City 970374, Taiwan

**Keywords:** rheumatoid arthritis, autoantibodies, histones, systemic lupus erythematosus, Sjögren disease

## Abstract

**Background/Objectives**: To compare the positivity rates and serum levels of antibodies against histone H3 lysine 9 (anti-H3K9) and its lactylated form (anti-H3K9la) among patients with rheumatoid arthritis (RA), systemic lupus erythematosus (SLE), or Sjögren disease (SjD) and controls. **Methods**: Serum anti-H3K9 and anti-H3K9la levels were measured by enzyme-linked immunosorbent assay from 93 patients with RA, 40 with SLE, 40 with SjD, and 40 controls. Antibody findings were compared across study groups and according to rheumatoid factor immunoglobulin M (RF-IgM) and anti-citrullinated protein antibody (ACPA) status in RA. **Results:** Anti-H3K9 positivity was 28.0% in RA, 40.0% in SLE, 0% in SjD, and 2.5% in controls. Anti-H3K9la positivity was 22.6% in RA and 20.0% in SLE; no SjD patients or controls were positive. Patients with SjD had lower levels of both antibodies than controls. No statistically significant differences were detected according to RF-IgM or ACPA status, although the seronegative subgroups were small. In exploratory multivariable models, TNF inhibitor use was associated with higher levels of both antibodies. Adding either antibody to ACPA and RF-IgM increased sensitivity at the expense of specificity. **Conclusions:** Anti-H3K9 and anti-H3K9la antibodies were detected more frequently in RA and SLE than in controls, whereas patients with SjD had lower serum levels of both antibodies. No statistically significant differences in antibody positivity or levels were detected according to RF-IgM or ACPA status in RA, although the small seronegative subgroups limited the precision of these comparisons. Their potential value as adjunctive diagnostic biomarkers requires confirmation in larger, independent cohorts with formal diagnostic-performance analyses.

## 1. Introduction

Rheumatoid arthritis (RA) is a systemic inflammatory disease characterized primarily by synovitis and progressive joint damage and is associated with substantial morbidity and excess mortality [1]. The 2010 American College of Rheumatology (ACR)/European League Against Rheumatism (EULAR) classification criteria incorporate joint involvement, symptom duration, acute-phase reactants, rheumatoid factor (RF), and anti-citrullinated protein antibodies (ACPAs) [2]. Traditionally, patients with RA often received treatment with disease-modifying antirheumatic drugs (DMARDs) such as methotrexate (MTX) and steroid to stop joint inflammation. If treatment goal is not reached, biologic agents including tumor necrosis factor (TNF) inhibitors, interleukin-6 (IL-6) inhibitor, T cell costimulation inhibitor, B-cell depleting agents or targeted synthetic (ts) DMARDs should be considered [3]. Some biologic agents can alter circulating autoantibody levels in patients with RA [4]. Biologic therapies are also used in juvenile idiopathic arthritis (JIA) [5], and childhood-onset arthritis may remain active into adulthood [6].

The presence of serum autoantibodies is associated with diagnosis, disease activity, joint damage, and extra-articular manifestations of RA [7,8]. In addition, several autoantibodies were found to participate in the pathogenesis of RA [9,10]. The identification of additional autoantibodies therefore remains an active area of RA research. In addition to RF and ACPAs, anti-carbamylated protein antibodies (anti-CarP) [11], anti-acetylated protein antibodies (AAPAs) [12], anti-malondialdehyde-acetaldehyde antibodies (anti-MAA) [13], and antibodies against peptidylarginine deiminases (anti-PAD antibodies) [14] have been reported in RA. Many of these antibody cluster with established seropositivity, leaving RF- and ACPA-negative RA a continuing diagnostic challenge [15]. Distinguishing ACPA-negative RA from diseases that may present with inflammatory polyarthritis, including systemic lupus erythematosus (SLE) and Sjögren disease (SjD), can therefore be difficult and may delay diagnosis [15,16]. Wu et al. recently reported antibodies against lactylated histones in RA and found that antibodies against lactylated histone H3 lysine 9 (anti-H3K9la) were associated with disease activity and occurred in both ACPA-positive and ACPA-negative patients [17].

We hypothesized that antibodies against histone H3 lysine 9 (anti-H3K9) and its lactylated form might help distinguish RA from SLE and SjD. This study compared the positivity rates and serum levels of anti-H3K9 and anti-H3K9la antibodies among patients with RA, SLE, or SjD and controls. We also examined whether these antibodies were associated with RF-IgM or ACPA status, disease activity, and current antirheumatic therapy in patients with RA.

## 2. Materials and Methods

### 2.1. Study Design and Participants

This cross-sectional study included 93 patients with RA who fulfilled the 2010 American College of Rheumatology (ACR)/European League Against Rheumatism (EULAR) classification criteria for RA [2], 40 patients with SLE who fulfilled the 2019 EULAR/ACR classification criteria [18], 40 patients with SjD who fulfilled the 2016 ACR/EULAR classification criteria [19], and 40 controls. Patients who had previously received rituximab were excluded because B-cell depletion can reduce circulating autoantibody levels [4]. Serum samples and clinical data for the RA group were obtained from the Human Biobank of Dalin Tzu Chi Hospital, Buddhist Tzu Chi Medical Foundation, with approval from its Ethics and Governance Council (approval number: BKOUT115-1). Serum samples from participants with SLE and SjD were collected after written informed consent. RA disease activity was assessed using the 28-joint Disease Activity Score based on the erythrocyte sedimentation rate (DAS28-ESR). The study protocol was approved by the Human Research Ethics Committee of Dalin Tzu Chi Hospital, Buddhist Tzu Chi Medical Foundation (No. B11501020). The study was conducted in accordance with the Declaration of Helsinki.

### 2.2. Measurement of Anti-H3K9 and Anti-H3K9la Antibodies

Serum anti-H3K9 and anti-H3K9la antibodies were measured using an in-house enzyme-linked immunosorbent assay adapted, with modifications, from Wu et al. [17]. Synthetic histone H3 peptide corresponding to amino acids 4–13 KQTARKSTGG and the corresponding H3K9-lactylated peptide with amino acids 4–13 KQTARKSTGG (K represents a lysine residue modified by lactylation) were purchased from NovoPro Bioscience (Shanghai, China). Microplates (BD Biosciences, San Jose, CA, USA) were coated with 5 μg/mL H3K9 or H3K9la peptide in carbonate coating buffer containing 35 mM sodium bicarbonate and 15 mM sodium carbonate (pH 9.6) for 2 h at 37 °C. After four washes with phosphate-buffered saline (PBS), the plates were blocked overnight with 2% skim milk in PBS. Quanta Lite reagents (Inova Diagnostics, San Diego, CA, USA) were then used according to the manufacturer’s instructions. Absorbance was measured at 450 nm using a SpectraMax iD5 multimode microplate reader (Molecular Devices, San Jose, CA, USA). Each plate included a standard curve generated from serial dilutions of serum from one patient with RA who had a high optical-density value. Antibody levels were expressed in assay units per milliliter (U/mL). Among the 40 controls, the Shapiro–Wilk tests did not detect a statistically significant departure from normality for anti-H3K9 (W = 0.960, *p* = 0.167) or anti-H3K9la (W = 0.961, *p* = 0.177). Positivity cutoffs were defined separately for anti-H3K9 and anti-H3K9la as the control-group mean plus two standard deviations. The intra-assay and inter-assay coefficient of variation were 5.3% and 6.2%, respectively.

### 2.3. Measurement of RF-IgM and ACPA

RF-IgM was measured using the EliA RF IgM Well on a Phadia 250 analyzer (Thermo Fisher Scientific, Waltham, MA, USA). ACPA was measured using a cyclic citrullinated peptide antibody enzyme-linked immunosorbent assay kit (Abnova, Taipei, Taiwan). Both assays were performed according to the manufacturers’ instructions.

### 2.4. Sensitivity and Specificity Analysis of Individual Antibodies and Antibody Combinations

For exploratory diagnostic-performance analyses, sensitivity was calculated among patients with RA, and specificity was calculated among non-RA participants, including patients with SLE or SjD and controls. For antibody combinations, a positive result was defined as positivity for at least one of the listed markers.

### 2.5. Statistical Analysis

Continuous variables are presented as mean ± standard deviation, and categorical variables as number (percentage). Between-group comparisons were performed using Student’s *t* test, the Mann–Whitney U test, the chi-square test, or Fisher’s exact test, as appropriate. Comparisons of demographic, disease-activity, and treatment characteristics according to anti-H3K9 and anti-H3K9la positivity were exploratory. Holm’s step-down procedure was applied separately to the nine comparisons of demographic, disease-activity, and treatment characteristics according to anti-H3K9 positivity and the nine corresponding comparisons according to anti-H3K9la positivity. Both unadjusted and Holm-adjusted *p*-values are reported. Other exploratory comparisons and regression estimates were not adjusted for multiplicity and require confirmation. Univariable linear regression was used to examine associations of age, sex, DAS28-ESR, and medication use with anti-H3K9 and anti-H3K9la levels. Multivariable models included age, sex, DAS28-ESR, and tumor necrosis factor (TNF) inhibitor use. Regression coefficients are reported with 95% confidence intervals. All tests were two-sided, and *p* < 0.05 was considered statistically significant. Analyses were conducted using Small Stata 13.0 (StataCorp, College Station, TX, USA).

## 3. Results

### 3.1. Participant Characteristics

Participant characteristics are summarized in Table 1. Sex distribution did not differ significantly between the disease groups and controls. Patients with SLE were younger than controls and patients with SjD were older than controls. In the RA group, 66 of 93 patients (71.0%) were RF-IgM positive and 67 (72.0%) were ACPA positive. RF-IgM positivity was present in 4 of 40 patients with SLE (10.0%), 10 of 40 with SjD (25.0%), and 3 of 40 controls (7.5%). ACPA positivity was present in 1 of 40 patients with SLE (2.5%) and in none of the patients with SjD or controls.

### 3.2. Anti-H3K9 and Anti-H3K9la Antibodies Across Study Groups

**Figure 1:**
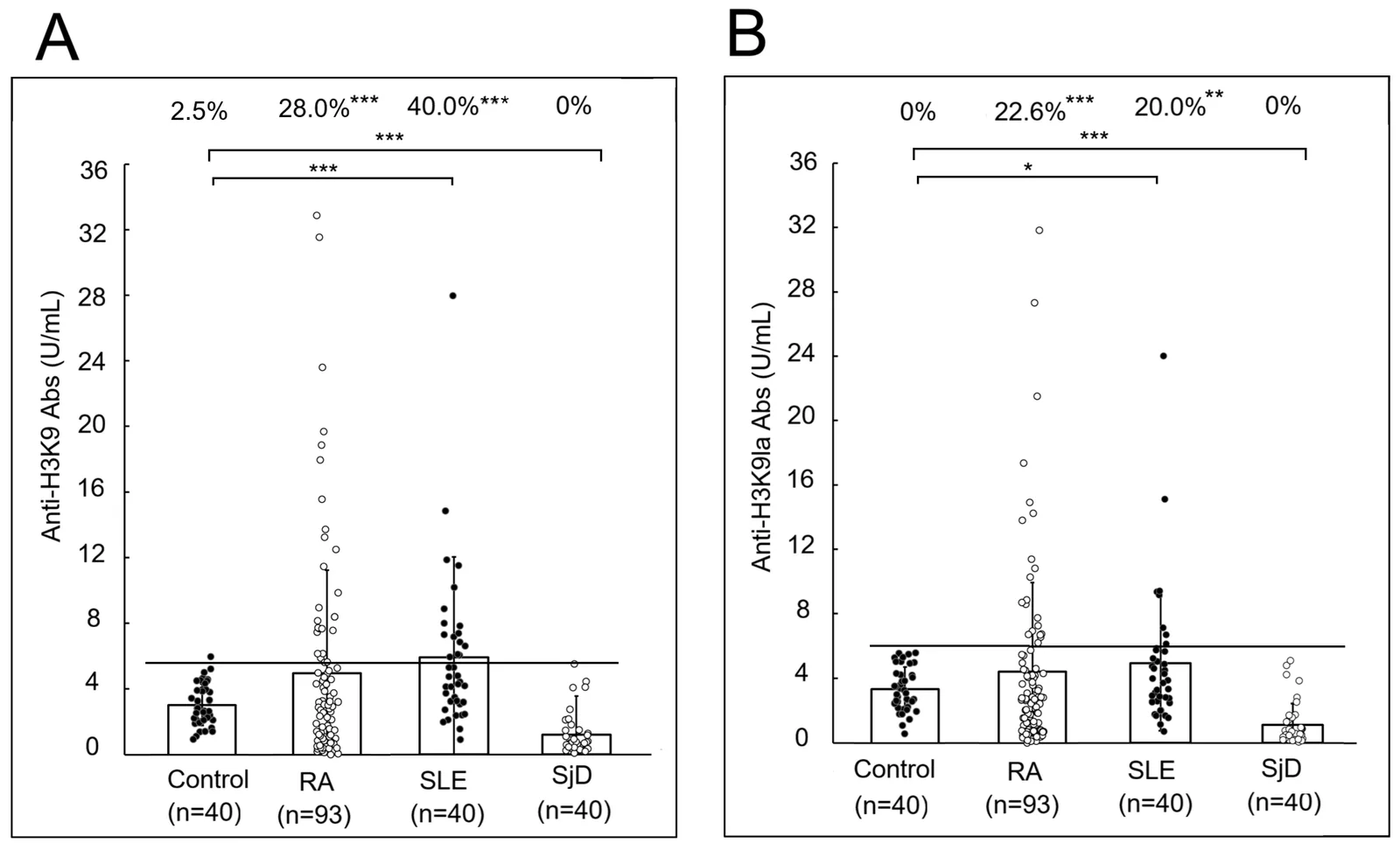
Positivity rates and serum levels of anti-H3K9 and anti-H3K9la antibodies among controls and patients with rheumatoid arthritis (RA), systemic lupus erythematosus (SLE), or Sjögren disease (SjD). (**A**) Anti-H3K9 antibody. (**B**) Anti-H3K9la antibody (* *p* < 0.05; ** *p* < 0.01; *** *p* < 0.001).

Anti-H3K9 positivity was more frequent in RA than in controls (28.0% vs. 2.5%, *p* < 0.001) and in SLE than in controls (40.0% vs. 2.5%, *p* < 0.001) (Figure 1A). No patient with SjD was anti-H3K9 positive. Mean anti-H3K9 levels did not differ between RA and controls (4.9 ± 6.3 vs. 3.0 ± 1.3 U/mL, *p* = 0.058), but were higher in SLE (5.9 ± 4.7 U/mL, *p* < 0.001) and lower in SjD (1.2 ± 1.3 U/mL, *p* < 0.001) than in controls. Anti-H3K9la positivity was observed in 22.6% of patients with RA and 20.0% of those with SLE, compared with 0% of controls (*p* < 0.001 and *p* = 0.005, respectively); no patient with SjD was positive (Figure 1B). Mean anti-H3K9la levels did not differ significantly between RA and controls (4.3 ± 5.4 vs. 3.3 ± 1.3 U/mL, *p* = 0.382) but were higher in SLE (4.9 ± 4.2 vs. 3.3 ± 1.3 U/mL, *p* = 0.021), and lower in SjD than in controls (1.1 ± 1.3 vs. 3.3 ± 1.3 U/mL, *p* < 0.001).

### 3.3. Antibody Findings According to RF-IgM and ACPA Status in RA

**Figure 2:**
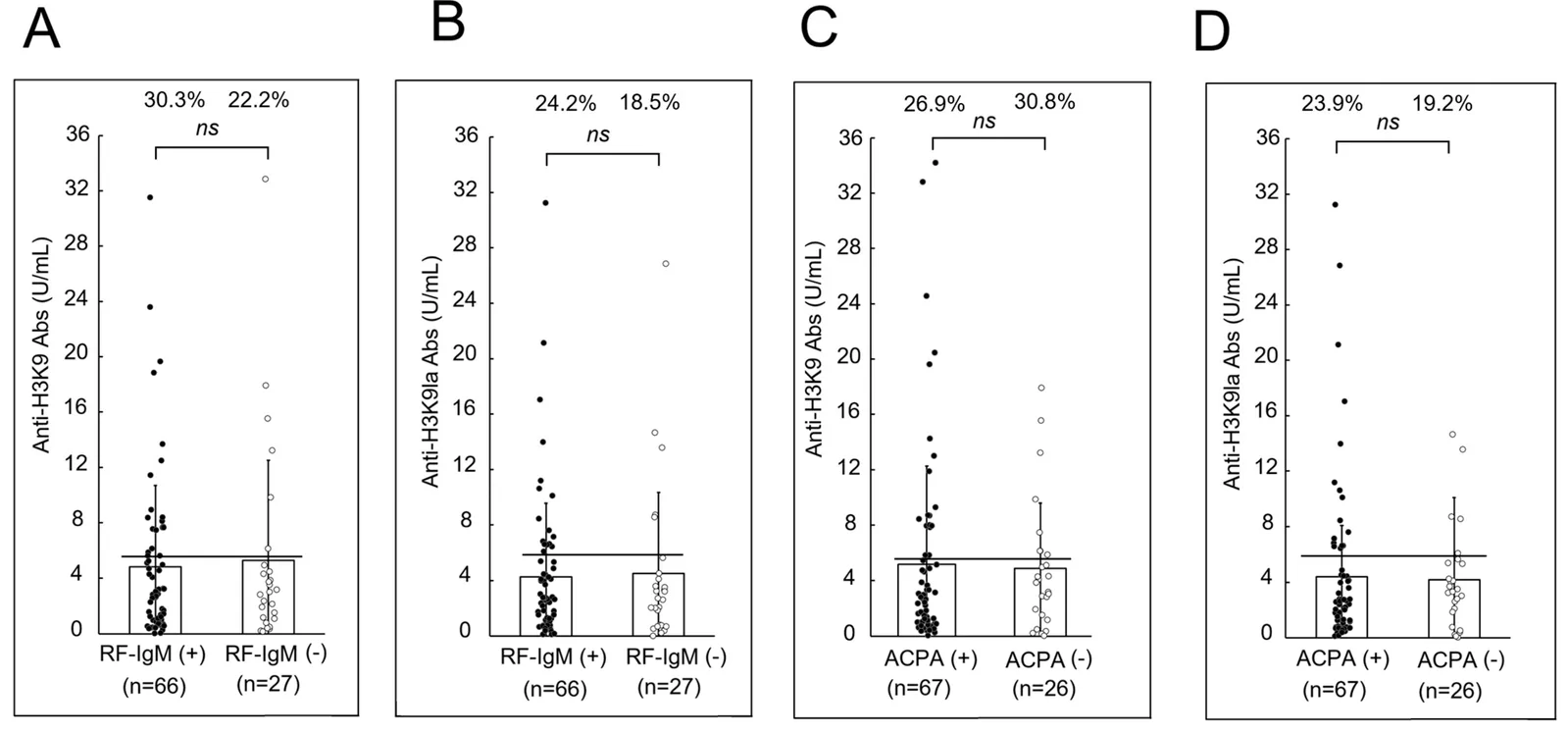
Positivity rates and serum levels of anti-H3K9 and anti-H3K9la antibodies in patients with rheumatoid arthritis (RA), stratified by rheumatoid factor immunoglobulin M (RF-IgM) or anti-citrullinated protein antibody (ACPA) status. (**A**) Anti-H3K9 antibody by RF-IgM status. (**B**) Anti-H3K9la antibody by RF-IgM status. (**C**) Anti-H3K9 antibody by ACPA status. (**D**) Anti-H3K9la antibody by ACPA status. ns: not significant.

Among patients with RA, anti-H3K9 positivity was observed in 30.3% of RF-IgM-positive patients and 22.2% of RF-IgM-negative patients (Figure 2A); corresponding anti-H3K9la positivity rates were 24.2% and 18.5% (Figure 2B). Anti-H3K9 positivity was observed in 26.9% of ACPA-positive patients and 30.8% of ACPA-negative patients (Figure 2C); corresponding anti-H3K9la positivity rates were 23.9% and 19.2% (Figure 2D). Neither antibody positivity nor serum levels differed significantly according to RF-IgM or ACPA status.

### 3.4. Characteristics According to Anti-H3K9 Status

In the unadjusted comparisons, age, sex, and DAS28-ESR did not differ significantly between anti-H3K9-positive and anti-H3K9-negative patients with RA (Table 2). TNF inhibitor use was more frequent among anti-H3K9-positive patients (57.70% vs. 31.4%, *p* = 0.032). In contrast, interleukin-6 inhibitor use was significantly less frequent among anti-H3K9-positive patients (0% vs. 16.4%, *p* = 0.031). No comparison in Table 2 remained significant after Holm adjustment.

### 3.5. Characteristics According to Anti-H3K9la Status

All anti-H3K9la-positive patients were women; the proportion of men was lower in the anti-H3K9la-positive group than in the negative group (0% vs. 18.1%, *p* = 0.036). The anti-H3K9la-positive group also had a higher mean DAS28-ESR (3.37 ± 0.70 vs. 2.81 ± 1.08, *p* = 0.028) (Table 3). Medication use did not differ significantly between the groups. No comparison in Table 3 remained significant after Holm adjustment.

### 3.6. Clinical Correlates of Antibody Levels in RA

In univariate analyses, male sex was associated with a lower anti-H3K9 level (regression coefficient, −3.82 U/mL; 95% confidence interval [CI], −7.47 to −0.17), and TNF inhibitor use was associated with higher anti-H3K9 and anti-H3K9la levels (Table 4). In multivariable models adjusted for age, sex, and DAS28-ESR, TNF inhibitor use remained associated with higher anti-H3K9 (coefficient, 3.03 U/mL; 95% confidence interval, 0.48–5.58) and anti-H3K9la levels (coefficient, 2.63 U/mL; 95% CI, 0.41–4.84).

### 3.7. Sensitivity and Specificity of Individual Antibodies and Antibody Combinations

Among the individual antibodies, ACPA showed the highest sensitivity for RA (72.0%), followed by RF-IgM (71.0%), anti-H3K9 (28.0%), and anti-H3K9la (22.6%). ACPA also showed the highest specificity (99.2%), compared with 85.8% for RF-IgM, 85.8% for anti-H3K9, and 93.3% for anti-H3K9la (Table 5) for distinguishing rheumatoid arthritis from non-rheumatoid arthritis participants. Combining ACPA with RF-IgM increased sensitivity to 81.7%, although specificity decreased to 85.8%. Adding anti-H3K9 to this combination further increased sensitivity to 87.1%, with a corresponding decrease in specificity to 74.2%. When anti-H3K9la was added instead, sensitivity was 86.0% and specificity was 80.0%. The specificities of individual antibodies and antibody combinations for distinguish RA from controls, SLE or SjD participants were list in Table 5.

## 4. Discussion

In this cross-sectional cohort, anti-H3K9 and anti-H3K9la antibodies were detected more frequently in patients with RA or SLE than in controls. Anti-H3K9 antibodies are a type of anti-histone antibody (AHA). AHAs are uncommon and generally present at low levels in patients with RA [20]. A previous study associated AHAs with Felty’s syndrome, which is now a rare complication of RA [21]. We did not find this association in our study. In exploratory multivariable models, TNF inhibitor use was associated with higher anti-H3K9 and anti-H3K9la levels; these models were not adjusted for multiplicity. Previous studies have also reported associations between AHA positivity or levels and TNF inhibitor use, although they included relatively few patients and lacked controls [22,23,24]. TNF inhibitor-associated autoantibody formation is a possible explanation for the observed antibody elevations [25,26]. The association in our cross-sectional study may also reflect treatment indication or other patient characteristics, and these data cannot distinguish a treatment effect from disease-related antibody reactivity or confounding by indication. In the comparisons stratified by antibody positivity, the nominal associations of anti-H3K9 positivity with TNF inhibitor and IL-6 inhibitor use, and of anti-H3K9la positivity with higher DAS28-ESR and sex, did not remain statistically significant after Holm adjustment. These findings should therefore be interpreted as exploratory. Liang et al. reported that an anti-IL-6 antibody reduced anti-dsDNA autoantibodies in a murine lupus model [27], and Wu et al. reported an association between anti-H3K9la and RA disease activity [17]. These reports provide context for the nominal findings, but our adjusted results do not establish the corresponding associations.

We found that the positivity rates of anti-H3K9 and anti-H3K9la antibodies and the serum levels of anti-H3K9 antibodies were significantly higher in patients with SLE than in controls. AHAs are frequently found in patients with SLE [28]. AHAs include antibodies against all histone subunits, including H1, H2A, H2B, H3, and H4, and correlate with interferon-induced inflammatory biomarkers in patients with SLE [28,29,30]. Our findings are broadly consistent with previous reports of antihistone antibodies in SLE. Some immune abnormalities occur across rheumatic diseases, as illustrated by increased proportions of αβTCR+ double-negative T cells in pediatric patients with different rheumatic conditions [31]. RA and SLE also exhibit NET-related immune dysregulation [32] and shared gene-expression signatures associated with immune responses and inflammation [33]. The presence of anti-H3K9 and anti-H3K9la antibodies in patients with RA and SLE is not unexpected. In contrast, patients with SjD had very low positivity and lower levels of both peptide-specific antibodies than controls, despite a previous report of anti-histone antibodies in 21.6% of patients with primary SjD [34]. Complete absence of positivity in SjD is unusually and further studies are needed. SLE and RA are frequently associated with secondary Sjögren’s syndrome. Testing serum anti-H3K9 or anti-H3K9la antibody levels might help differentiate SLE or RA from SjD.

Anti-H3K9 and anti-H3K9la antibodies were detected in both RF-IgM-positive and RF-IgM-negative RA and in both ACPA-positive and ACPA-negative RA. We did not detect statistically significant differences in antibody positivity or levels according to RF-IgM or ACPA status. This finding is consistent with that of Wu et al. [17]. Approximately 20–30% of patients with RA are seronegative for conventional autoantibodies, whereas ACPA, RF, anti-CarP antibodies, AAPAs, and anti-PAD antibodies frequently coexist in seropositive RA. Patients with seronegative RA may therefore be more difficult to classify using current criteria, supporting the search for additional biomarkers that provide information beyond ACPA and RF status [15]. Grönwall et al. reported higher Anti-MAA levels in patients with ACPA-positive RA than in those with ACPA-negative RA. However, the difference in the proportion of patients with elevated IgG anti-MAA levels was no longer statistically significant after adjustment for sex, age, and DAS28 [35]. We noted that there are 17 RF-IgM and ACPA-negative RA patients. Among these patients, five are positive for anti-H3K9 or four are positive for anti-H3K9la. However, because the RF-IgM-negative, ACPA-negative and RF-IgM and ACPA-negative subgroups were relatively small, these null findings should not be interpreted as evidence that anti-H3K9 or anti-H3K9la antibodies are independent of conventional RA serostatus. The presence of these antibodies in some seronegative patients raises the possibility of additional serologic information, but this requires confirmation in substantially larger cohorts enriched for RF- and ACPA-negative RA.

In the diagnostic-performance analysis, combining ACPA with RF-IgM yielded a sensitivity of 81.7% and a specificity of 85.8% for distinguishing RA from non-RA participants. Adding anti-H3K9 increased sensitivity to 87.1%, with specificity decreasing to 74.2%, whereas adding anti-H3K9la yielded a sensitivity of 86.0% and a specificity of 80.0%. Thus, the addition of either antibody increased sensitivity at the expense of specificity. The reduction in specificity may partly reflect the composition of the non-RA group, which included 40 patients with SLE, a disease in which anti-histone antibodies are common and in which anti-H3K9 and anti-H3K9la positivity was also observed in our study. It should be caution that higher sensitivity does not necessarily mean better overall diagnostic performance. In contrast, Wu et al. found little anti-lactylated histone autoantibody reactivity in patients with psoriatic arthritis, another clinically relevant differential diagnosis for RA [17]. Diagnostic performance may therefore vary according to the spectrum of comparator diseases. The incremental value of anti-H3K9 and anti-H3K9la, particularly in patients with seronegative RA, requires further evaluation in larger, independent cohorts with clinically relevant comparator groups.

This study has several limitations. The cross-sectional design precludes assessment of temporal or causal relationships, and the association with TNF inhibitor use may reflect confounding by indication or other patient characteristics. Complete information on previous TNF inhibitor exposure was unavailable, precluding a sensitivity analysis restricted to patients with documented absence of previous exposure. The treated, single-center RA population may limit generalizability to untreated patients and other populations. Only one participant in our RA cohort had a history of JIA. We therefore could not draw conclusions about anti-H3K9 and anti-H3K9la antibody profiles or their associations with biologic treatment in patients with JIA. Differences in age across disease groups may have affected the between-group comparisons and were not resolved by the RA-only regression models. The seronegative subgroups were small, so null comparisons do not establish independence from RF-IgM or ACPA status. The positivity cutoffs were derived from 40 controls, who also contributed to specificity estimation, and have not been independently validated. Antibodies were measured using an in-house assay with participant samples assayed once and relative calibration based on serum from one patient. These features limit assay standardization and interpretation near the positivity threshold. The analyses were exploratory; Holm adjustment applied to Table 2 and Table 3 does not address multiplicity in the other comparisons or regression models. The diagnostic-performance findings therefore require confirmation in independent cohorts with validated assays, prespecified cutoffs, and clinically relevant comparator groups.

## 5. Conclusions

Anti-H3K9 and anti-H3K9la antibodies were detected more frequently in RA and SLE than in controls, whereas patients with SjD had lower serum levels of both antibodies. Within RA, no statistically significant differences were detected according to RF-IgM or ACPA status, although the small seronegative subgroups limited precision. Nominal associations of antibody positivity with sex, disease activity, and treatment use did not remain statistically significant after Holm adjustment. In separate exploratory multivariable models without multiplicity adjustment, TNF inhibitor use was associated with higher antibody levels; the cross-sectional design precludes causal interpretation. Adding either antibody to ACPA and RF-IgM increased sensitivity at the expense of specificity. These findings do not establish improved overall diagnostic performance, and the potential adjunctive value of these antibodies requires validation in larger, independent cohorts.

## Figures and Tables

**Table 1:** Characteristics of controls and patients with rheumatoid arthritis, systemic lupus erythematosus, and Sjögren disease.

Variable	Controls (*n* = 40)	Rheumatoid Arthritis (*n* = 93)	Systemic Lupus Erythematosus (*n* = 40)	Sjögren Disease (*n* = 40)
Age, years	56.8 ± 8.6	60.2 ± 10.2	42.0 ± 12.6 *	61.9 ± 12.4 *
Sex, female: male	32:8	80:13	33:7	37:3
RF-IgM positive	3 (7.5)	66 (71.0)	4 (10.0)	10 (25.0)
ACPA positive	0 (0)	67 (72.0)	1 (2.5)	0 (0)

Values are mean ± standard deviation or *n* (%). ACPA, anti-citrullinated protein antibody; RF-IgM, rheumatoid factor immunoglobulin M. * *p* < 0.05 versus controls.

**Table 2:** Characteristics of patients with rheumatoid arthritis according to anti-H3K9 antibody status.

Variable	Anti-H3K9 Positive (*n* = 26)	Anti-H3K9 Negative (*n* = 67)	*p*-Value	Holm- Adjusted *p*-Value
Sex, male	1 (3.8)	12 (17.9)	0.102	0.714
Age, years	59.6 ± 10.8	60.5 ± 10.0	0.637	>0.999
DAS28-ESR	3.16 ± 0.88	2.85 ± 1.08	0.193	>0.999
**Medication**				
Prednisolone-equivalent dose, mg/day	4.8 ± 1.6	4.8 ± 1.8	0.938	>0.999
Methotrexate	21 (80.8)	55 (82.1)	>0.999	>0.999
TNF inhibitor	15 (57.7)	21 (31.4)	0.032	0.279
Interleukin-6 inhibitor	0 (0)	11 (16.4)	0.031	0.279
T cell costimulation inhibitor	0 (0)	2 (3.01)	>0.999	>0.999
Targeted synthetic DMARD	2 (7.7)	10 (14.9)	0.499	>0.999

Values are mean ± standard deviation or *n* (%). DAS28-ESR, 28-joint Disease Activity Score based on erythrocyte sedimentation rate; DMARD, disease-modifying antirheumatic drug; TNF, tumor necrosis factor.

**Table 3:** Characteristics of patients with rheumatoid arthritis according to anti-H3K9la antibody status.

Variable	Anti-H3K9la Positive (*n* = 21)	Anti-H3K9la Negative (*n* = 72)	*p*-Value	Holm- Adjusted *p*-Value
Sex, male	0 (0.0)	13 (18.1)	0.036	0.288
Age, years	62.1 ± 8.6	59.6 ± 10.6	0.321	>0.999
DAS28-ESR	3.37 ± 0.70	2.81 ± 1.08	0.028	0.252
**Medication**				
Prednisolone-equivalent dose, mg/day	5.0 ± 1.1	4.7 ± 1.9	0.526	>0.999
Methotrexate	17 (81.0)	59 (81.9)	>0.999	>0.999
TNF inhibitor	11 (52.4)	25 (34.7)	0.203	>0.999
Interleukin-6 inhibitor	0 (0)	11 (15.3)	0.064	0.448
T cell costimulation inhibitor	0 (0)	2 (2.8)	>0.999	>0.999
Targeted synthetic DMARD	2 (9.5)	10 (13.9)	0.728	>0.999

Values are mean ± standard deviation or *n* (%). DAS28-ESR, 28-joint Disease Activity Score based on erythrocyte sedimentation rate; DMARD, disease-modifying antirheumatic drug; TNF, tumor necrosis factor.

**Table 4:** Univariate and multivariable linear regression analyses of clinical factors associated with anti-H3K9 and anti-H3K9la levels in rheumatoid arthritis.

Variable	Univariable Analysis Coefficient (95% CI)		Multivariable Analysis Coefficient (95% CI)	
	Anti-H3K9	Anti-H3K9la	Anti-H3K9	Anti-H3K9la
Sex, male vs. female	−3.82 * (−7.47, −0.17)	−3.16 (−6.33, 0.01)	−3.16 (−6.81, 0.49)	−2.56 (−5.73, 0.61)
Age, per 10 years	0.50 (−0.78, 1.78)	0.57 (−0.54, 1.68)	0.47 (−0.77, 1.71)	0.56 (−0.52, 1.63)
DAS28-ESR	1.17 (−0.07, 2.41)	0.96 (−0.12, 2.03)	0.82 (−0.41, 2.05)	0.66 (−0.40, 1.73)
Prednisolone-equivalent dose, mg/day	0.34 (−0.39, 1.08)	0.28 (−0.36, 0.92)	–	–
Methotrexate, yes vs. no	0.63 (−2.72, 3.98)	1.00 (−1.90, 3.90)	–	–
TNF inhibitor, yes vs. no	3.07 * (0.49, 5.65)	2.62 * (0.38, 4.86)	3.03 * (0.48, 5.58)	2.63 * (0.41, 4.84)
Interleukin-6 inhibitor, yes vs. no	−3.40 (−7.35, 0.55)	−2.79 (−6.21, 0.64)	–	–
T-cell costimulation inhibitor, yes vs. no	−3.49 (−12.39, 5.41)	−3.01 (−10.73, 4.70)	–	–
Targeted synthetic DMARD, yes vs. no	−1.95 (−5.80, 1.89)	−1.65 (−4.98, 1.68)	–	–

Coefficients are expressed in U/mL. CI, confidence interval; DAS28-ESR, 28-joint Disease Activity Score based on erythrocyte sedimentation rate; DMARD, disease-modifying antirheumatic drug; TNF, tumor necrosis factor. * *p* < 0.05.

**Table 5:** Sensitivity and specificity of individual antibodies and antibody combinations for distinguishing rheumatoid arthritis from controls, SLE, SjD or non-rheumatoid arthritis participants in the study cohort.

Marker or Positivity Criterion	Sensitivity, % (95% CI)	Specificity, % (Controls; 95% CI)	Specificity, % (SLE; 95% CI)	Specificity, % (SjD; 95% CI)	Specificity, % (Non-RA; 95% CI)
**Individual markers**					
ACPA	72.0 (62.2, 80.1)	100.0 (91.2–100.0)	97.5 (87.1–99.6)	100.0 (91.2–100.0)	99.2 (95.4, 99.9)
RF-IgM	71.0 (61.1, 79.2)	92.5 (80.1–97.4)	90.0 (77.0–96.0)	75.0 (59.8–85.8)	85.8 (78.5, 91.0)
anti-H3K9	28.0 (19.9, 37.8)	97.5 (87.1–99.6)	60.0 (44.6–73.7)	100.0 (91.2–100.0)	85.8 (78.5, 91.0)
anti-H3K9la	22.6 (15.3, 32.1)	100.0 (91.2–100.0)	80.0 (65.2–89.5)	100.0 (91.2–100.0)	93.3 (87.4, 96.6)
**Two-marker combinations**					
ACPA or RF-IgM positive	81.7 (72.7, 88.3)	92.5 (80.1–97.4)	90.0 (77.0–96.0)	75.0 (59.8–85.8)	85.8 (78.5, 91.0)
ACPA or anti-H3K9 positive	79.6 (70.3, 86.5)	97.5 (87.1–99.6)	60.0 (44.6–73.7)	100.0 (91.2–100.0)	85.8 (78.5, 91.0)
ACPA or anti-H3K9la positive	77.4 (67.9, 84.7)	100.0 (91.2–100.0)	77.5 (62.5–87.7)	100.0 (91.2–100.0)	92.5 (86.4, 96.0)
**Three-marker combinations**					
ACPA, RF-IgM or anti-H3K9 positive	87.1 (78.8, 92.5)	90.0 (77.0–96.0)	57.5 (42.2–71.5)	75.0 (59.8–85.8)	74.2 (65.7, 81.2)
ACPA, RF-IgM or anti-H3K9la positive	86.0 (77.5, 91.7)	92.5 (80.1–97.4)	75.0 (59.8–85.8)	75.0 (59.8–85.8)	80.0 (72.0, 86.2)

For antibody combinations, a positive result was defined as positivity for at least one of the listed markers. ACPA, anti-citrullinated protein antibody; RF-IgM, immunoglobulin M rheumatoid fac tor; H3K9, histone H3 lysine 9; H3K9la, lactylated histone H3 lysine 9.

## Data Availability

The datasets analyzed during the current study are available from the corresponding author on reasonable request.

## Abbreviations

The following abbreviations are used in this manuscript:

AAPAs	Anti-acetylated protein antibodies
ACPA	Anti-citrullinated protein antibody
ACR	American College of Rheumatology
Anti-CarP	Anti-carbamylated protein antibodies
Anti-H3K9	Antibodies against histone H3 lysine 9
Anti-H3K9la	Antibodies against lactylated histone H3 lysine 9
Anti-MAA	Anti-malondialdehyde-acetaldehyde antibodies
Anti-PAD	Antibodies against peptidylarginine deiminases
DAS28-ESR	28-joint Disease Activity Score based on the erythrocyte sedimentation rate
DMARDs	Disease-modifying antirheumatic drugs
EULAR	European League Against Rheumatism
IL-6	Interleukin-6
MTX	Methotrexate
PBS	Phosphate-buffered saline
RA	Rheumatoid arthritis
RF	Rheumatoid factor
RF-IgM	Rheumatoid factor immunoglobulin M
SjD	Sjögren disease
SLE	Systemic lupus erythematosus
TNF	Tumor necrosis factor
tsDMARD	Targeted synthetic disease-modifying antirheumatic drugs
